# Glycine propionyl-L-carnitine increases plasma nitrate/nitrite in resistance trained men

**DOI:** 10.1186/1550-2783-4-22

**Published:** 2007-12-03

**Authors:** Richard J Bloomer, Webb A Smith, Kelsey H Fisher-Wellman

**Affiliations:** 1 Department of Health and Sport Sciences, University of Memphis, Memphis, TN, USA

## Abstract

We have recently demonstrated that oral intake of glycine propionyl-L-carnitine (GPLC) increases plasma nitrate/nitrite (NOx), a surrogate measure of nitric oxide production. However, these findings were observed at rest, and in previously sedentary subjects.

In the present study, we sought to determine the impact of oral GPLC on plasma NOx at rest and in response to a period of reactive hyperemia in resistance trained men.

Using a double blind, crossover design, 15 healthy men (24 ± 4 years) were assigned to GPLC (3 g/d PLC + 1044 mg glycine) and a placebo in random order, for a four-week period, with a two-week washout between condition assignment. Blood samples were taken from subjects at rest and at 0, 3, and 10 minutes following an ischemia-reperfusion protocol (six minutes of upper arm cuff occlusion at 200 mmHg followed by rapid reperfusion with cuff removal). Blood samples were taken from a forearm vein from the same arm used for the protocol and analyzed for total nitrate/nitrite. Data are presented as mean ± SEM.

A condition main effect (p = 0.0008) was noted for NOx, with higher values in subjects when using GPLC (45.6 ± 2.8 μmol·L^-1^) compared to placebo (34.9 ± 1.2 μmol·L^-1^). No time main effect was noted (p = 0.7099), although values increased approximately 12% from rest (37.7 ± 2.7 μmol·L^-1^) to a peak at 10 minutes post protocol (42.3 ± 3.3 μmol·L^-1^). The interaction effect was not significant (p = 0.8809), although paired time contrasts revealed higher values for GPLC compared to placebo at 3 (48.2 ± 6.7 vs. 34.9 ± 2.4 μmol·L^-1^; p = 0.033) and 10 (48.8 ± 5.9 vs. 35.7 ± 2.1 μmol·L^-1^; p = 0.036) minutes post protocol, with non-statistically significant differences noted at rest (41.8 ± 4.5 vs. 33.6 ± 2.5 μmol·L^-1^; p = 0.189) and at 0 minutes (43.6 ± 5.1 vs. 35.4 ± 2.7 μmol·L^-1^; p = 0.187) post protocol. An analysis by subject (collapsed across time) indicated that 11 of the 15 subjects experienced an increase in NOx with GPLC treatment.

These findings indicate that short-term oral GPLC supplementation can increase NOx in resistance trained men. However, as with many dietary supplements, there exist both "responders" and "non-responders" to treatment. Future work may focus on the mechanisms for the discrepancy in response to GPLC supplementation for purposes of NOx elevation.

## Introduction

Nitric oxide (NO^•^) is an important signaling molecule, promoting vasodilation by acting on vascular smooth muscle [1]. In addition, NO^• ^has been linked to other physiological functions such as inhibition of platelet aggregation and platelet adhesion [2]. In these ways, NO^• ^mediates increased blood flow at rest [3-5] and during exercise [4,6,7], which may have implications for those with chronic ischemic disease as well as for athletes interested in enhancing blood flow to working skeletal muscle (e.g., endurance athletes, bodybuilders).

Nitric oxide is synthesized from L-arginine by the endothelium in the vascular system [2] and has been reported to increase in response to acute exercise [8-13]. This is true for dynamic [4], as well as for isometric [7] exercise, in which reperfusion is present following cessation of the contraction. Moreover, chronic exercise training has been reported to result in an increase in plasma nitrate/nitrite (NOx) levels [14-16], a surrogate marker of NO^•^.

Aside from exercise, pharmaceutical agents have been used with success to induce NO^• ^biosynthesis, in an attempt to promote vasodilation (for review please see [17]). Treatments have sometimes included high dosages of L-arginine, often provided via intravenous injection. This has led to the development of several nutritional supplements, often containing small amounts of L-arginine, which are purported to have "drug-like" action, ultimately leading to increased NO^•^. While the fitness industry is inundated with advertisements for such products, the scientific data are scant. We have recently demonstrated an increase in plasma NOx in healthy sedentary subjects following oral treatment with a novel carnitine agent, glycine propionyl-L-carnitine (GPLC; [18]). This finding extends recent work of Lofreddo et al. [19] who reported an increase in blood NOx in response to 6 grams per day of PLC given via IV infusion to patients with peripheral arterial disease. Other reports suggest vasodilatation actions with PLC [20], in addition to glycine [21] treatment independently. However, our initial findings using oral GPLC were observed at rest, and in previously sedentary subjects, individuals who quite possibly have lower resting NOx levels compared to well-trained subjects; hence, may have a greater potential for responding to GPLC treatment. To our knowledge, no studies have been done to evaluate the efficacy of nutritional supplements to increase NOx levels in a sample of potential users (e.g., resistance trained athletes). Therefore, in the present study, our purpose was to determine the impact of oral GPLC on plasma NOx at rest and in response to a period of reactive hyperemia in resistance trained men. Using a double blind, randomized, crossover design, we hypothesized that plasma NOx would be higher with GPLC treatment compared to placebo, at rest and in response to the ischemia-reperfusion protocol.

## Methods

### Subjects

Fifteen healthy, resistance trained men participated in this investigation. Subjects completed a medical history, diet and supplementation history, and physical activity questionnaire to determine eligibility. No subject was a smoker or used anti-inflammatory drugs or antioxidant supplements before (for a minimum of six months) or during the study period. Subjects were young (24 ± 4 yrs; mean ± SD), and of average height (177 ± 5 cm), weight (83 ± 4 kg), and body fat percentage (14 ± 5 %). Subjects were considered to be well-trained and performed resistance exercise for 5 ± 2 hrs per week for 8 ± 5 yrs. They were instructed not to deviate from their current training regimen during the course of the study with the exception of refraining from exercise for the 48 hours prior to each testing day. All experimental procedures were performed in accordance with the Helsinki Declaration. The University of Memphis Human Subjects Committee approved all experimental procedures. All subjects provided both verbal and written consent prior to participating in this study.

### Conditions

Subjects were randomly assigned in double-blind manner using a cross-over design to GPLC and a placebo. Each intake period consisted of four weeks, with a two-week washout period between condition assignments. Subjects were instructed to ingest a total of six capsules per day of GPLC or placebo (at two separate times – morning and evening). Based on recent evidence indicating that carnitine uptake is enhanced with carbohydrate feeding [22,23], subjects were instructed to take capsules along with a carbohydrate rich meal. GPLC is a USP grade nutritional supplement as of March 2006, consisting of a molecular bonded form of propionyl-L-carnitine (PLC) and the amino acid glycine (GlycoCarn™, Sigma-tau HealthScience S.p.A, Rome, Italy). In the total 4.5 g/d dosage of GPLC, the actual PLC content was equal to 3 g and the glycine content was equal to 1044 mg (the remainder of the capsule consisted primarily of cellulose). The short term (8–9 week) safety of GPLC supplementation at the dosage provided (4.5 g/d) was established in our initial work [18], where we noted no adverse changes in subjects' complete blood count or blood chemistry panel data. Capsules were manufactured by Jarrow Formulas (Los Angeles, CA), were identical in appearance, and were provided to subjects in unlabeled bottles every two weeks. Compliance to intake was determined based on capsule counts upon bottle return.

### Ischemia reperfusion protocol

Following each four-week period of either GPLC or placebo intake, subjects underwent a protocol involving five minutes of upper arm cuff occlusion in conjunction with submaximal isometric handgrip dynamometry to induce ischemia. The tests were conducted on day 29, following the four-week treatment period. The time of testing was matched for placebo and GPLC conditions for all subjects, and all testing was done between 0600–0900 hours. All subjects reported to the lab for testing, on both days, in a 10-hour fasted state. No food was allowed until after the 10-min post protocol blood draw. A standard blood pressure cuff was applied to the dominant upper arm and inflated to 200 mmHg. This is a common procedure used in the study of brachial artery blood flow in response to various stimuli, which results in enhanced blood flow and artery dilation during the reperfusion period, mediated by NO^• ^[24-27]. Hence, we used this protocol to study the plasma NOx response. At the end of the five minute period, subjects performed an isometric contraction using a handgrip dynamometer at 50% of their maximal predetermined capacity, for one minute. Therefore, the total duration of cuff occlusion was six minutes. At the end of the six minute period, the pressure in the cuff was immediately removed to allow for rapid reperfusion (reactive hyperemia). Blood samples were collected before, immediately post (0), 3 min post, and 10 min post protocol, as described below.

### Blood collection and biochemistry

Venous blood samples (~7 mL) were taken from subjects via needle and EDTA containing vacutainer by a trained phlebotomist. Following collection, blood samples were placed on ice and immediately centrifuged at 1500 × g at 4°C for 15 min. Plasma was then removed and immediately stored at -80°C until assayed for nitrate/nitrite (NOx). Nitrate/nitrite was analyzed using commercially available assay kits (Caymen Chemical, Ann Arbor, MI). Following conversion of nitrate to nitrite using nitrate reductase, Greiss reagent was added to the sample, which converts nitrite into a deep purple azo compound. The absorbance of this azo chromophore was then detected photometrically at 540 nm using a BioTek Instruments Powerwave 340 microplate reader (Winooski, Vermont), and unknown sample values were determined using a nitrite standard curve, via software (BioTek Gen5™). Assays were performed in duplicate and the coefficient of variation was 5.1%.

### Dietary records

Subjects were instructed to maintain their normal diet during the study period and to complete food records to allow for nutrient intake assessment for the seven days prior to each testing session. Subjects were given specific instructions regarding the recording of portion sizes and quantities, in addition to viewing food models in order to enhance precision. Diet records were analyzed for total calories, protein, carbohydrate, and fat (Food Processor SQL, version 9.9, ESHA Research, Salem, OR).

### Statistical analysis

Plasma NOx data were analyzed using a 2 (condition) × 4 (time) repeated measures analysis of variance (ANOVA). Single degree of freedom contrasts were used to determine condition differences at each time. Such a priori contrasts do not require adjustment of the type I error rate. Dietary data were analyzed using a one way ANOVA. These data are presented as mean ± standard error of the mean. Subject descriptive characteristics are presented as mean ± standard deviation. All analyses were performed using JMP statistical software (version 4.0.3, SAS Institute, Cary, NC). Statistical significance was set at P ≤ 0.05.

## Results

Of the 16 subjects who started the study, only data from 15 subjects were included in the analysis. One subject failed to return to the lab for the second testing session; therefore, data for this subject were excluded. Compliance to GPLC and placebo intake was ≥ 97% for both conditions based on capsule counting upon return of bottles. Subjects indicated their compliance to our recommendation of maintenance of their specific exercise regimen during the course of the study period. The mean daily intake of kilocalories (2750 ± 195 vs. 2801 ± 217; p = 0.86), protein grams (155 ± 14 vs. 157 ± 15; p = 0.91), carbohydrate grams (330 ± 23 vs. 340 ± 34; p = 0.76), and fat grams (87 ± 9 vs. 81 ± 7; p = 0.60) during the week prior to GPLC and placebo conditions, respectively, did not differ. Analyses of diet records indicated that no subject regularly consumed nitrate- (e.g., spinach, cabbage, beets, radishes) or nitrite-rich foods during the diet reporting periods; therefore, we do not believe that dietary intake influenced the plasma NOx values.

**Figure 1:**
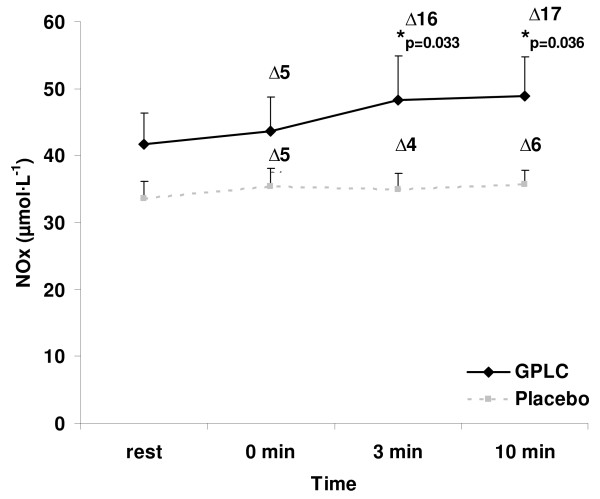
**Plasma nitrate/nitrite before and after an ischemia-reperfusion protocol in 15 resistance trained men supplemented with GPLC and placebo in a cross-over design**. Note: Condition main effect (p = 0.0008); No time main effect (p = 0.7099) or interaction effect (p = 0.8809); paired time contrasts at 3 (p = 0.033) and 10 (p = 0.036) minutes post protocol; rest (p = 0.189) and 0 (p = 0.187) minutes post protocol; % change from rest presented for each time post protocol. Values are mean ± SEM.

**Figure 2:**
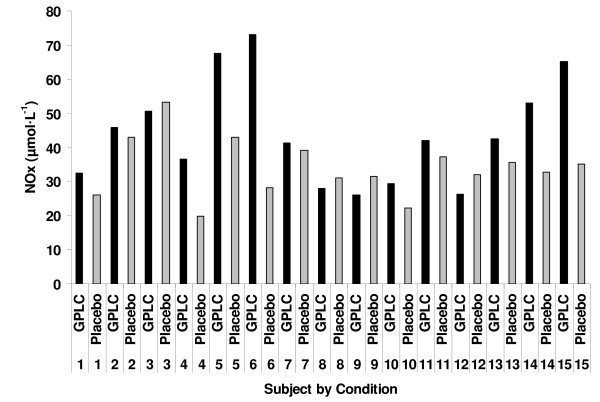
**Individual subject data for plasma nitrate/nitrite before and after an ischemia-reperfusion protocol in 15 resistance trained men supplemented with GPLC and placebo in a cross-over design**. Note: NOx data collapsed over time.

A condition main effect (p = 0.0008) was noted for NOx, with higher values in subjects when using GPLC (45.6 ± 2.8 μmol·L^-1^) compared to placebo (34.9 ± 1.2 μmol·L^-1^). No time main effect was noted for NOx (p = 0.7099), although values increased approximately 12% from rest to a peak at 10 minutes post protocol. The interaction effect for NOx was not significant (p = 0.8809), although paired time contrasts revealed higher values for GPLC compared to placebo at 3 (p = 0.033) and 10 (p = 0.036) minutes post protocol. The interaction data for NOx are presented in Figure 1. An analysis by subject (collapsed across time) indicated that 11 of the 15 subjects experienced an increase in NOx with GPLC treatment (Figure 2).

## Discussion

We report for the first time that a nutritional supplement, in the form of oral GPLC, can increase plasma NOx in resistance trained men. These findings may have implications for enhanced blood flow to active skeletal muscle during exercise. This may be important for endurance athletes, for whom increased blood flow may aid exercise performance [15,28], as well as for bodybuilders, for whom increased blood flow may lead to greater delivery of substrates (e.g., amino acids) during recovery, which may increase protein synthesis [29].

Although we noted no time main effect or interaction effect of NOx (Figure 1), values were generally higher at all times following the protocol, with a peak increase from rest at 10 minutes post protocol. Furthermore, although no interaction effect was noted, values were statistically higher with the GPLC treatment compared to placebo at 3 and 10 minutes post protocol, with non-statistically higher values noted at rest and at 0 minutes post protocol. Therefore, while resting NOx levels may not be increased significantly with GPLC treatment in resistance trained men, NOx in response to an acute physical stimulus appears higher with GPLC treatment compared to placebo. For example, the increase from rest with GPLC was 16% and 17% at 3 and 10 minutes post protocol, respectively. Whereas, the increase from rest with placebo was only 4% and 6% at 3 and 10 minutes post protocol, respectively (Figure 1). Although these findings are interesting, future work should include flow mediated dilation studies as a compliment to the plasma NOx measurement, in order to investigate bloodflow specific changes due to GPLC treatment. This would provide insight into the physiological relevance of the NOx increase.

Despite our findings that the majority of subjects responded to GPLC treatment (11 of 15; Figure 2), there were those individuals who were "non-responders". This is common with nutritional supplements, and with regards to dietary carnitine supplementation, may be related to carbohydrate intake. That is, based on the recent findings of Stephens and coworkers [22,23], carbohydrate rich feedings, which induce a state of hyperinsulinemia, appear to improve carnitine transport into skeletal muscle. Hence, subjects with a low carbohydrate intake, in particular as related to the actual time of carnitine ingestion, may experience less than optimal uptake into tissue. This may also be true as related to uptake into endothelial cells. If this were the case, it is possible that the effects of GPLC on NOx would be minimized. However, we analyzed the mean daily carbohydrate intake of all subjects and noted no difference between the carbohydrate intake of "responders" (342 ± 27 g) and "non-responders (316 ± 31 g). Moreover, we noted no correlation between carbohydrate intake and NOx levels (p = 0.27). Therefore, other mechanisms must be involved related to these subjects' inability to respond to GPLC treatment. Future study is needed to explore the potential mechanisms associated with this finding. In addition, the specific mechanisms responsible for the increase in plasma NOx with GPLC supplementation remain to be elucidated. That is, in vitro data indicate that maintenance of NO^• ^with PLC treatment may be associated with decreased NADPH oxidase activation [30], an enzyme which subsequently leads to superoxide radical generation [31] and decreased NO^• ^bioavailability. Moreover, recent work indicates that PLC augments endothelial nitric oxide synthase (eNOS) [32], the major enzyme responsible for NO^• ^production. Clearly, these findings provide some mechanistic evidence to support our data; however more work is needed to more fully understand the mechanisms associated with the increase in plasma NOx (perhaps investigation of second messenger systems such as cGMP) with GPLC supplementation in human subjects.

## Conclusion

In summary, our findings indicate that short-term oral GPLC supplementation can increase NOx in resistance trained men. However, there exist both "responders" and "non-responders" to treatment. Findings from this study may have health implications for those with ischemic conditions such as peripheral vascular disease and ischemic heart disease, as increased NOx may allow for enhanced blood flow, in particular during times of physical stress. Moreover, these findings may relate specifically to athletes who seek enhanced blood flow during periods of strenuous exercise, as such a change may be associated with improved physical performance during training and optimal post exercise recovery. Future study is needed to determine what, if any, physiologic benefit is associated with the observed increase in plasma NOx with GPLC supplementation.

## Competing interests

The author(s) declare that they have no competing interests.

## Authors' contributions

RB was responsible for the study design, biochemical work, statistical analyses, and manuscript preparation; WS was responsible for the study design, data collection, blood collection and processing, and manuscript preparation. KFW was responsible for data collection, blood collection and processing, and manuscript preparation. All authors read and approved of the final manuscript.
